# Tri-State Meeting

**Published:** 1898-07

**Authors:** 


### Tri-State Meeting.

At the meeting of the Tri-State body at Put-in-Bay, the State meetings of Michigan, Ohio and Indiana were present. Michigan and Indiana each held its State meeting, which took the place of the annual meeting, the usual time for both these being in June. These two bodies convened and accomplished the routine business of their annual meeting, electing officers, receiving new members, appointing delegates to the National Dental Association, etc., etc.

The Michigan Society elected for its President, Dr. C. B. Blackmarr; Secretary, Dr. G. S. Dennis; Treasurer, Dr. G. H. Moser.

The Indiana State Society also did the routine business for its annual meeting, electing for its President for the coming year Dr. A. Jameson, of Indianapolis; Secretary, ——. Several new members were elected, and delegates appointed to the National Dental Association.

The Ohio State Society also held a session for any routine business that might come before it. This meeting does not, however, take the place of its regular annual meeting, as was the case with the other States, but this will be held at the usual time, the first Tuesday in December, 1898, in Columbus. Delegates were elected to the National Dental Association.

